# Feeding Strategy Shapes the Evolution of the Hyaluronidase Gene Family in Medicinal Leeches

**DOI:** 10.1002/ece3.74049

**Published:** 2026-07-14

**Authors:** Rujiao Sun, Xingke Fu, Rui Ai, Zuhao Huang, Huanhuan Li, Lizhou Tang, Huanhuan Chen, Xiongsheng Zhang, Fang Zhao, Gonghua Lin

**Affiliations:** ^1^ School of Life Sciences, Key Laboratory of Jiangxi Province for Biological Invasion and Biosecurity Jinggangshan University Ji'an China; ^2^ College of Life Sciences Jiangxi Normal University Nanchang China; ^3^ Yunnan International Joint Laboratory With South and Southeast Asia for the Integrated Development of Animal‐Derived Anti‐Thrombosis Chinese Medicine Qujing Normal University Qujing China; ^4^ Nanning Zhitai Biological Research Co., Ltd. Nanning China

**Keywords:** feeding strategy, gene family evolution, hematophagy, hyaluronidase, leech

## Abstract

Hyaluronidase plays a critical role as a “spreading factor” during blood‐feeding in leeches, yet the molecular evolution of this gene family remains poorly understood. In this study, we combined phylogenetic reconstruction, selective pressure analyses, and transcriptome‐based expression profiling to investigate the evolutionary characteristics of the hyaluronidase gene family across eight medicinal leech species representing hematophagous and non‐hematophagous lineages. Our results showed that all hematophagous species retained five complete hyaluronidase gene family members (*hya1*–*hya5*), whereas non‐hematophagous species lacked *hya5*. Phylogenetic analyses revealed that each gene family member formed a well‐supported monophyletic clade, indicating that ancestral gene duplications occurred prior to species divergence. Although all hyaluronidase genes remained under strong purifying selection (*ω* < 1), hematophagous lineages exhibited significantly higher *ω* values than non‐hematophagous lineages, suggesting relatively relaxed selective constraints in hematophagous lineages. Transcriptome analyses further demonstrated that hyaluronidase genes were significantly more highly expressed in hematophagous leeches, with *hya1* and *hya2* consistently showing the highest expression levels. Overall, our findings suggest that feeding strategy is closely associated with the retention, selective constraint, and transcriptional regulation of the hyaluronidase gene family in the leech species examined here, providing new insights into the molecular evolution of blood‐feeding‐related genes.

## Graphical abstract

We investigated the molecular evolution and expression divergence of the hyaluronidase gene family across eight medicinal leech species with contrasting feeding strategies. Hematophagous species retained a complete five‐member gene repertoire and exhibited elevated *dN*/*dS* values and significantly elevated gene expression compared to non‐hematophagous taxa. These results suggest that feeding strategy has played a key role in shaping gene copy number variation, evolutionary constraints, and expression divergence in this gene family.

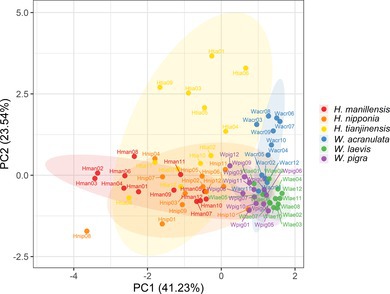

**Abbreviations**
BEB: Bayes empirical Bayes
BIC: Bayesian information criterion
CDSs: coding sequences
CNGBdb: China National GeneBank DataBase
ECM: extracellular matrix
GH79: glycoside hydrolase family 79
hya1–hya5: hyaluronidase gene family members 1–5
LDTI: leech‐derived tryptase inhibitor
NGDC: National Genomics Data Center
RNA‐Seq: transcriptome sequencing
TPM: transcripts per million

## 1 Introduction

Leeches, as an ancient group of annelids, have been used for medical purposes throughout both Eastern and Western history. In China, their medicinal value was first systematically documented in *Shennong's Classic of Materia Medica*, where they were primarily used for the treatment of disorders associated with blood stasis (Wu et al. 2024; Wang et al. 2021). In Western medicine, historical records ranging from therapeutic scenes depicted in ancient Egyptian tomb murals to the widespread practice of bloodletting in medieval Europe demonstrate their long‐standing role as an important medical tool (Whitaker et al. 2004; Kirk and Pemberton 2011). Modern scientific investigation into leech‐derived bioactive substances began in 1884, when Haycraft first reported anticoagulant activity in leech extracts (Haycraft 1884). The anticoagulant principle was subsequently termed ‘hirudin’ by Jacoby in 1904, and hirudin was first successfully isolated as a purified protein by Markwardt (1955). This work marked an important milestone and stimulated subsequent research into leech‐derived bioactive compounds. The therapeutic value of leeches is mainly attributed to the diverse bioactive components present in their saliva, including hirudin, which exhibits the strongest known anticoagulant activity; Eglin C, an inhibitor of elastase and neutrophil proteinases; antistasin, a specific inhibitor of coagulation factor Xa; and decorsin, a specific antagonist of the platelet glycoprotein IIb–IIIa receptor (Seymour et al. 1990; Seemüller et al. 1980; Dunwiddie et al. 1989). Acting synergistically, these components exert potent antithrombotic effects, thereby establishing the importance of leeches in both traditional medicine and modern research on thrombotic diseases (Montinari and Minelli 2022).

However, leeches comprise a large number of species, and their bioactive components vary substantially among different taxa. Taking hirudin as an example, no hirudin has been identified in *Whitmania acranulata* (Whitman, 1886) or *Whitmania laevis* (Baird, 1869) (Zhao et al. 2025), whereas five and seven hirudin members have been detected in *Hirudinaria manillensis* (Lesson, 1842) and *Whitmania pigra* (Whitman, 1884), respectively. Nevertheless, among the five hirudin members identified in *H. manillensis* , only three exhibit significant anticoagulant activity, whereas only one of the seven hirudin members identified in *W. pigra* shows anticoagulant activity (Liu et al. 2023, 2024; Müller et al. 2022). In addition, even between closely related species such as *Hirudo nipponia* (Whitman, 1886) and *Hirudo tianjinensis* (Wang, 2022), which possess the same number of hirudin genes, substantial differences in expression levels have been observed (Yin et al. 2025). Similarly, previous studies have shown that most leech species, including *H. manillensis* , *Hirudo medicinalis* (Linnaeus, 1758), *H. nipponia*, *H. tianjinensis*, *Whitmania pigra*, and *W. laevis*, harbor only a single member of the leech‐derived tryptase inhibitor (LDTI) family, whereas *W. acranulata* contains six LDTI members. Despite this difference in gene copy number, the expression levels of LDTIs are significantly higher in hematophagous leeches (Xiao et al. 2025). Therefore, to systematically evaluate the molecular evolutionary characteristics of leech bioactive components and the factors influencing their diversification, it is necessary to select species with broad taxonomic representation.

At present, both domestic and international studies on leech bioactive components have predominantly focused on substances with direct antithrombotic effects. For example, hirudin (Markwardt 1955) and antistasin (Nutt et al. 1988) function as anticoagulants, destabilase (Marin et al. 2023) and hementerin (Chudzinski‐Tavassi et al. 1998) exhibit thrombolytic activity, and decorsin (Seymour et al. 1990) inhibits platelet aggregation. However, leeches also possess another class of bioactive molecules that do not directly target the host coagulation system but instead facilitate the diffusion and effectiveness of these antithrombotic components. These molecules play a crucial role in leech survival and are also of significant importance for the development of leech‐derived therapeutic products. Nevertheless, they have received far less attention in current research. A representative and functionally important example is hyaluronidase.

Hyaluronidases are a class of hydrolases that degrade hyaluronic acid in the extracellular matrix (ECM) and are therefore commonly referred to as typical “spreading factors” due to their ability to facilitate the diffusion of other molecules within tissues (Lu et al. 2025). During blood feeding, hyaluronic acid in host tissues constitutes an important physical barrier. Leech‐secreted hyaluronidase hydrolyzes hyaluronic acid, markedly reducing tissue viscoelasticity and thereby accelerating the penetration of anticoagulants and other bioactive molecules, which enhances feeding efficiency and overall pharmacological effects (Claude 1937). From an enzymatic classification perspective, hyaluronidases can be divided into three major types: hyaluronoglucosaminidase (EC 3.2.1.35), hyaluronoglucosidase (EC 3.2.1.36), and hyaluronate lyase (EC 4.2.2.1) (Meyer 1971). Leech hyaluronidases belong to the hyaluronoglucosidase category and are classified within the glycoside hydrolase family 79 (GH79) (Lv et al. 2016; Linker et al. 1957). Compared with hyaluronidases derived from mammals and bacteria, leech hyaluronidases exhibit higher substrate specificity and a narrower product distribution, conferring distinct advantages in diffusion activity and potential therapeutic applications (Jin et al. 2014). Hyaluronidase not only represents a key factor enabling leeches to adapt to hematophagy but also constitutes an important molecular component in the long‐term co‐evolution between leeches and their hosts, and it plays a significant role in applications within the pharmaceutical and biomaterials fields (Lv et al. 2016; Kraemer et al. 1988; McGuire et al. 2024; Weber et al. 2019).

Although numerous studies have reported various genes and bioactive peptides in leeches (Liu et al. 2023; Ye et al. 2025; Zhao et al. 2024; Sun et al. 2025; Baskova et al. 2004), research on leech hyaluronidases remains very limited. Existing studies have primarily focused on the three‐dimensional structure and preparation of leech hyaluronidase (Jin et al. 2014; Huang et al. 2022; Liao et al. 2023), with little attention paid to the molecular evolution of this gene family across different leech species. Therefore, in this study, representative species from the genera *Hirudinaria*, *Hirudo*, and *Whitmania*—including *H. manillensis* , *Hirudinaria bpling* (Wahlberg, 1855), *H. medicinalis* , *H. nipponia*, *H. tianjinensis*, *W. acranulata*, *W. laevis*, and *W. pigra*—were selected. By integrating comparative genomics and transcriptomic data, we analyzed the phylogenetic relationships, selective pressures, and transcriptional expression patterns of hyaluronidase genes in leeches with different feeding strategies, with the aim of elucidating the molecular evolutionary characteristics of this gene family and the factors influencing these characteristics.

## 2 Materials and Methods

### 2.1 RNA Sequencing and Sequence Retrieval

This study focused on *H. manillensis* collected from Ding'an, Hainan (19°46′ N, 110°27′ E), and *H. nipponia*, *H. tianjinensis*, *W. acranulata*, *W. laevis*, and *W. pigra* collected from Baodi, Tianjin (39°43′ N, 117°30′ E). Sampling was conducted between May and July, coinciding with the peak activity period of leeches, in shallow freshwater habitats including ponds, paddy fields, and irrigation ditches. All individuals were wild‐caught and identified to species in the field based on morphological characteristics (Yang 1996). For each species, 12 individuals were randomly collected. Specimens were immediately dissected upon collection to remove digestive tract tissues. Total RNA was extracted from head tissues using TRIzol Reagent (Invitrogen, Carlsbad, CA, USA) and further purified with the RNeasy Mini Kit (Qiagen, Chatsworth, CA, USA). RNA samples passing quality control were used for library construction, with an average insert size of approximately 300 bp. Transcriptome sequencing (RNA‐Seq) was performed on an Illumina HiSeq platform, with each individual sequenced separately to generate 12 independent RNA‐seq libraries per species. Raw reads were quality‐filtered using fastp v0.20.0 (Chen et al. 2018) to generate clean reads, which were then de novo assembled into transcript (unigene) sequences using Trinity v2.9.0 (Grabherr et al. 2011). Raw RNA‐seq data used in this study have been deposited in the China National GeneBank DataBase (CNGBdb), which is maintained by the National Genomics Data Center (NGDC), under two BioProject accession numbers: PRJCA046291 and PRJCA058083.

Because live specimens of *H. medicinalis* and *H. bpling* were unavailable, publicly accessible data were used to complement the analyses. Hyaluronidase gene sequences of *H. medicinalis* were obtained from two GenBank genome assemblies (GCA_011800805.1 and GCA_903470615.1), whereas sequences for *H. bpling* were retrieved from BioProject PRJNA1120733, encompassing RNA‐Seq, Hi‐C, and PacBio datasets (Khan et al. 2025).

Considering that some hyaluronidase gene copies in leech genomes may be structurally incomplete or transcriptionally inactive, relying solely on genome alignments could result in incomplete identification of candidate genes. Therefore, transcriptome data were primarily used to screen for hyaluronidase homologs, focusing on sequences with clear transcriptional evidence. Previously reported hyaluronidase protein sequences served as references (Zhao et al. 2025, 2024; Liu et al. 2023, 2024; Babenko et al. 2020), and BLAST v2.13.0+ (Camacho et al. 2009) was employed to identify all potential homologous sequences within each species' unigene set. The resulting transcripts were mapped to the corresponding genomes, and Exonerate (protein2genome model) (Slater and Birney 2005) was used to verify exon–intron structures. Gene structure completeness was further confirmed by the presence of conserved GT–AG splice signals. Gene copies identified in the genome but lacking transcriptional evidence were excluded from downstream analyses to ensure that only transcriptionally supported and potentially functional gene copies were considered.

### 2.2 Sequence Variation and Phylogenetic Analysis

Based on the sequences obtained as described above, sequence variation analyses were performed for the hyaluronidase gene family members (*hya1*–*hya5*) across eight leech species: *H. manillensis* , *H. bpling*, *H. medicinalis* , *H. nipponia*, *H. tianjinensis*, *W. pigra*, *W. laevis*, and *W. acranulata*. All coding sequences (CDSs) used in this study are provided in Supporting Information S1. CDSs of each gene were aligned at the nucleotide level using MEGA 5 (Tamura et al. 2011) with the MUSCLE algorithm under default parameters. CDS length information was obtained directly from the corresponding FASTA files. Protein sequences were translated from the CDSs, and protein lengths were calculated based on the resulting amino acid sequences. Based on the CDS multiple sequence alignments, the aligned length and the number of variable sites were calculated for each gene. Pairwise sequence identity analyses were also conducted based on CDS nucleotide alignments, excluding alignment positions consisting solely of gaps. To avoid the influence of sequence differences among different hyaluronidase gene family members (*hya1*–*hya5*) on the analysis, conservation and similarity analyses were performed separately for each family member. Gene structure analyses were carried out using Exonerate with the protein2genome model (Slater and Birney 2005), in which protein sequences were accurately aligned to the corresponding genomic sequences. Exon–intron structural features, including intron positions and exon numbers, were inferred directly from the predicted splice sites in the alignment results.

Based on the sequence variation analyses described above, phylogenetic analyses of hyaluronidase genes were subsequently conducted. CDSs were first translated into amino acid sequences in MEGA 5 (Tamura et al. 2011) and aligned using the MUSCLE algorithm. Codon‐preserving nucleotide alignments were then generated based on the corresponding protein alignments. The resulting alignments were exported in FASTA format and used as input for phylogenetic analyses in IQ‐TREE v2.2.0 (Minh et al. 2020). Phylogenetic trees were reconstructed using the maximum likelihood method. The best‐fit substitution model was automatically selected using the ModelFinder module according to the Bayesian information criterion (BIC), and branch support was evaluated with 1000 ultrafast bootstrap replicates. *Haemadipsa yanyuanensis* (Lin et al. 2025) was designated as the outgroup. The outgroup sequence was identified using *H. bpling hya2*—the most conserved hyaluronidase paralog in our dataset—as a query sequence to retrieve the homologous gene from the *H. yanyuanensis* genome via sequence similarity search, yielding *H. yanyuanensis hya2*, which was used as the outgroup in the phylogenetic analysis. The resulting hyaluronidase gene phylogenetic trees were visualized and edited using FigTree v1.4.4 (Rambaut 2018) and Inkscape v1.2.2 (The Inkscape Team 2023).

### 2.3 Selection Pressure Analysis

To investigate differences in selective pressures acting on hyaluronidase genes between hematophagous and non‐hematophagous leeches, codon‐based selection pressure analyses were conducted using the CodeML module implemented in PAML‐X v1.2 (Xu and Yang 2013). Protein multiple‐sequence alignments and corresponding codon alignments were generated and manually inspected in MEGA using the standard genetic code (transl_table = 1).

Selective pressure analyses were performed using the two‐ratio branch model. Hematophagous leeches (*Hirudinaria* spp. and *Hirudo* spp.) were designated as foreground branches, whereas non‐hematophagous leeches (*Whitmania* spp.) together with the outgroup species *H. yanyuanensis* (Lin et al. 2025) and *Dinobdella ferox* (Gao et al. 2023) were treated as background branches. To avoid potential biases caused by combining paralogous genes with distinct evolutionary histories, each hyaluronidase gene member (*hya1*–*hya4*) was analyzed separately. *Hya5* was excluded from branch‐model comparisons because orthologous sequences were absent in non‐hematophagous species. Likelihood ratio tests (LRTs) were used to compare the two‐ratio model with the one‐ratio model (M0). The significance of model comparisons was evaluated using the chi‐square distribution with one degree of freedom.

### 2.4 Gene Expression Analysis

For each species, the index was constructed using the CDSs of the hyaluronidase genes identified in this study, as well as the CDSs of other genes previously annotated from the genomes (Liu et al. 2023, 2024; Müller et al. 2022; Baskova et al. 2004). RNA‐seq reads from each sample were mapped using Salmon v1.0.0 (Patro et al. 2017) with a k‐mer size of 31, and transcript abundance was quantified as transcripts per million (TPM) to assess relative gene expression levels. The mapping statistics for all RNA‐seq samples against the CDS reference datasets are summarized in Supporting Information S2 (Table S2). TPM data for all samples are provided in Supporting Information S3.

Differential expression analyses were performed using SPSS 26.0 (IBM Corp., Armonk, NY, USA) (IBM Corp 2019). The Shapiro–Wilk test indicated that expression data significantly deviated from a normal distribution (*p* < 0.001); therefore, non‐parametric statistical tests were applied. Overall expression levels between hematophagous and non‐hematophagous leeches were compared using the Mann–Whitney *U* test. Differences in expression among hyaluronidase gene family members, between hematophagous and non‐hematophagous leeches for each gene family member, and among members within species were assessed separately using the Kruskal–Wallis test, Mann–Whitney *U* test, and Friedman test, respectively.

To account for phylogenetic non‐independence among species, we performed phylogenetic ANOVA using the phylANOVA function in the R package phytools with 1000 permutations to compare expression levels between hematophagous and non‐hematophagous leeches. It should be noted that the non‐parametric tests described above (Mann–Whitney *U*, Kruskal–Wallis) do not account for phylogenetic relatedness and are only used for descriptive purposes; the primary statistical inferences for comparing hematophagous and non‐hematophagous leeches are based on the phylogenetic ANOVA. Visualization of expression data was conducted in RStudio v2024.12.1 (RStudio Team 2024) using R v4.4.2 (R Core Team 2024) and the ggplot2 package v3.5.2 (Wickham 2016), and bar plots were generated using GraphPad Prism 10.1.2 (GraphPad Software, San Diego, CA, USA) (GraphPad Software 2023). To evaluate expression patterns of the hyaluronidase gene family, clustering heatmaps were generated based on TPM values using TBtools‐II (Toolbox for Biologists) v2.327 (Chen et al. 2023), with Euclidean distance and the average linkage method. In addition, principal component analysis (PCA) was performed in R v4.4.2 (R Core Team 2024) using the prcomp() function. PCA scatter plots were visualized with the ggplot2 package v3.5.2 (Wickham 2016), with samples colored by species and confidence ellipses added to illustrate clustering patterns.

## 3 Results

### 3.1 Sequence Features and Structural Comparison of the Hyaluronidase Gene Family

Across the eight leech species examined, five hyaluronidase gene family members (*hya1*–*hya5*) were identified. All hematophagous leech species retained the full set of five members, whereas non‐hematophagous species lacked *hya5* and possessed only *hya1*–*hya4*. This difference reflects variation in hyaluronidase gene family composition between leeches with distinct feeding strategies (Table 1).

**TABLE 1:** Basic sequence features of hyaluronidase gene family members across different leech species.

Gene	Species	CDS length (bp)	Protein length (aa)	Exon number
*hya1*	*H. manillensis*	1509	503	10
*hya1*	*H. bpling*	1509	503	10
*hya1*	*H. medicinalis*	1521	507	10
*hya1*	*H. nipponia*	1521	507	10
*hya1*	*H. tianjinensis*	1521	507	10
*hya1*	*W. acranulata*	1566	522	10
*hya1*	*W. laevis*	1521	507	10
*hya1*	*W. pigra*	1521	507	10
*hya2*	*H. manillensis*	1509	503	10
*hya2*	*H. bpling*	1509	503	10
*hya2*	*H. medicinalis*	1506	502	10
*hya2*	*H. nipponia*	1509	503	10
*hya2*	*H. tianjinensis*	1509	503	10
*hya2*	*W. acranulata*	1509	503	10
*hya2*	*W. laevis*	1509	503	10
*hya2*	*W. pigra*	1509	503	10
*hya3*	*H. manillensis*	1458	486	10
*hya3*	*H. bpling*	1458	486	10
*hya3*	*H. medicinalis*	1455	485	10
*hya3*	*H. nipponia*	1458	486	10
*hya3*	*H. tianjinensis*	1458	486	10
*hya3*	*W. acranulata*	1455	485	10
*hya3*	*W. laevis*	1458	486	10
*hya3*	*W. pigra*	1458	486	10
*hya4*	*H. manillensis*	1515	505	11
*hya4*	*H. bpling*	1512	504	11
*hya4*	*H. medicinalis*	1506	502	11
*hya4*	*H. nipponia*	1512	504	11
*hya4*	*H. tianjinensis*	1512	504	11
*hya4*	*W. acranulata*	1512	504	11
*hya4*	*W. laevis*	1512	504	11
*hya4*	*W. pigra*	1512	504	11
*hya5*	*H. manillensis*	1530	510	11
*hya5*	*H. bpling*	1530	510	11
*hya5*	*H. medicinalis*	1527	509	11
*hya5*	*H. nipponia*	1527	509	11
*hya5*	*H. tianjinensis*	1527	509	11

The CDS lengths of hyaluronidase genes ranged from 1455 to 1566 bp, encoding proteins of 485–522 amino acids. CDS and protein lengths for the same gene were highly consistent across species, with only minor variations, indicating a high degree of structural conservation within this gene family. Gene structure analysis further revealed that *hya1*, *hya2*, and *hya3* each consisted of 10 exons, whereas *hya4* and *hya5* contained 11 exons. These exon–intron organizations were conserved across species, suggesting that gene structure is highly stable within the hyaluronidase gene family (Table 1).

Multiple sequence alignment showed that the aligned lengths of *hya1*–*hya5* ranged from 1458 to 1567 bp, with the number of variable sites ranging from 352 to 589 among different gene family members. It should be noted that the alignments in this study were constructed using homologous gene family members from different species; therefore, the relatively large number of variable sites primarily reflects evolutionary divergence among species rather than intraspecific polymorphism within a single species.

Pairwise sequence identity analysis indicated that hyaluronidase genes were highly conserved among closely related species, with sequence identities generally exceeding 95%. In contrast, sequence identities between more distantly related species were lower, typically ranging from approximately 72% to 90% (Table S1 in Supporting Information S2). This pattern of conservation was consistently observed across all hyaluronidase gene family members. Further comparison among different gene family members revealed variation in their pairwise sequence similarity ranges. Specifically, the sequence similarities of *hya1*, *hya2*, *hya3*, *hya4*, and *hya5* ranged from 82.82%–97.44%, 84.23%–99.01%, 81.24%–97.33%, 71.92%–98.28%, and 79.96%–95.62%, respectively. These results indicate that, although the hyaluronidase gene family is overall highly conserved, individual gene family members exhibit varying degrees of sequence divergence.

### 3.2 Phylogenetic Relationships of the Hyaluronidase Gene Family

**FIGURE 1:**
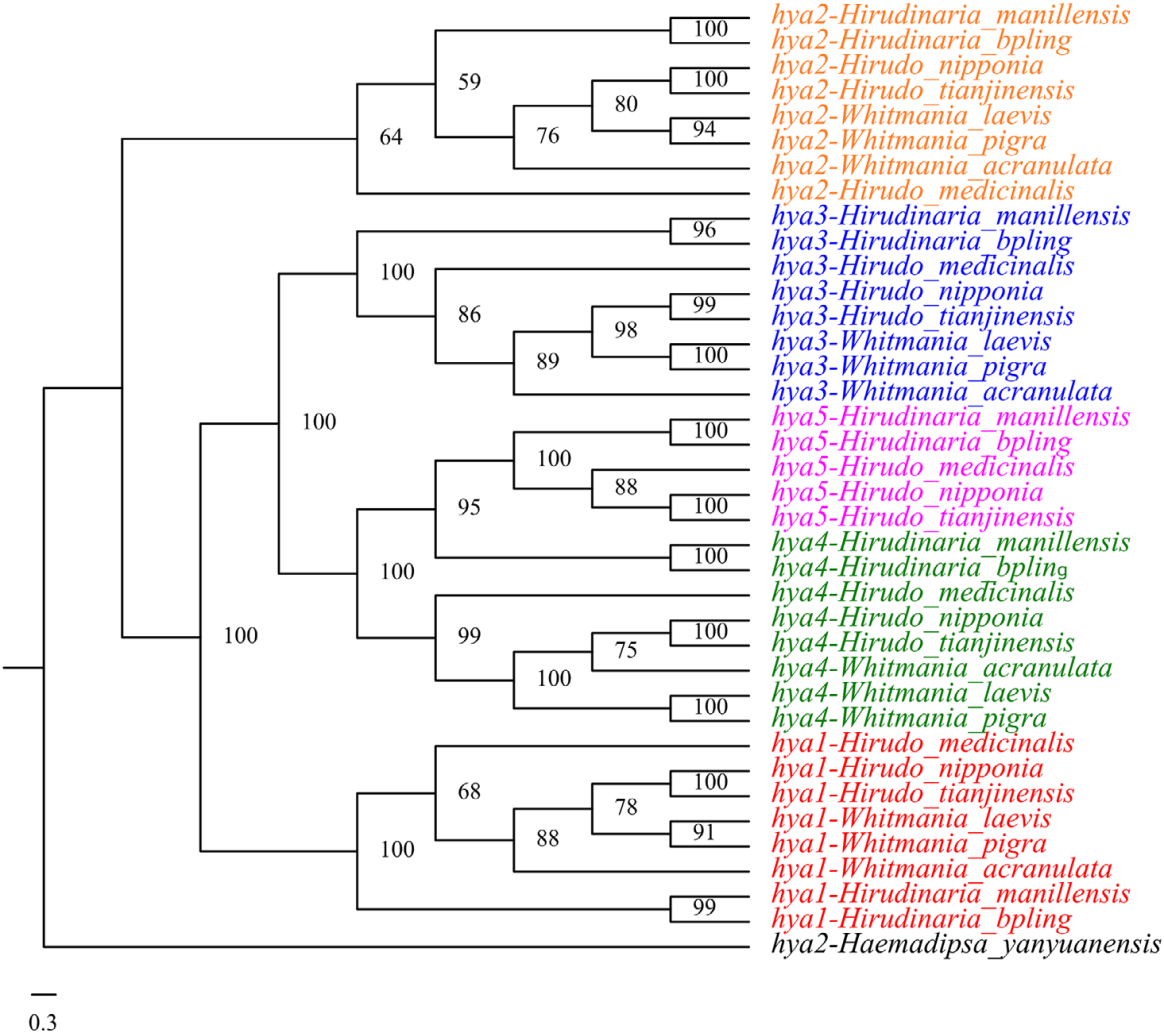
Phylogenetic tree of hyaluronidase genes across eight leech species. *H. yanyuanensis* was used as the outgroup. Different colors indicate the five major gene subgroups (*hya1*–*hya5*).

Based on the identification of hyaluronidase gene family members described above, we further examined the phylogenetic relationships and copy evolution of the hyaluronidase gene family. A phylogenetic tree was reconstructed using the hyaluronidase CDSs from eight leech species, with *H. yanyuanensis* designated as the outgroup. As shown in Figure 1, the hyaluronidase gene family was clearly resolved into five major clades corresponding to *hya1*–*hya5*, with each clade comprising orthologous gene copies from multiple species. Most clades were supported by bootstrap values exceeding 75%, indicating a robust and well‐supported gene tree topology. Most species retained multiple gene copies; however, patterns of gene copy retention across clades differed among species. Hematophagous species (*Hirudo* spp. and *Hirudinaria* spp.) possessed corresponding gene copies across all five clades (*hya1*–*hya5*), whereas non‐hematophagous *Whitmania* species lacked representative sequences in the *hya5* clade. In addition, a premature stop codon was detected in the *hya4* gene of *H. tianjinensis*, suggesting that this gene copy may represent a pseudogene.

Overall, each member of the hyaluronidase gene family formed a well‐supported monophyletic group, indicating that the diversification of hyaluronidase gene copies occurred prior to the speciation events of the leech lineages examined in this study. Subsequently, lineage‐specific patterns of gene retention and loss appear to have taken place during leech evolution.

### 3.3 Selection Pressure Analysis of the Hyaluronidase Gene Family

To investigate the evolutionary dynamics of the hyaluronidase gene family in leeches, codon‐based selection pressure analyses were performed using the two‐ratio branch model. Marked differences in *ω* values were observed among species (2Δln*L* = 33.64, *p* < 0.001), with a higher mean *ω* value in hematophagous lineages (0.198) than in background branches (0.119).

Gene‐specific analyses further revealed evolutionary differences among hyaluronidase paralogs (Table 2). Significant differences between hematophagous and non‐hematophagous lineages were detected for *hya2* (*p* < 0.001), *hya3* (*p* = 0.009), and *hya4* (*p* = 0.036), with consistently higher *ω* values observed in hematophagous lineages. In contrast, no significant difference was detected for *hya1* (*p* = 0.233), although it showed a slightly higher *ω* value in hematophagous lineages.

**TABLE 2:** Gene‐specific two‐ratio branch model analyses of selective pressures acting on hyaluronidase genes among different feeding lineages.

Gene	Foreground *ω*	Background *ω*	2Δln*L*	*p*
*hya1*	0.1893	0.1472	1.420	0.233
*hya2*	0.2041	0.0977	11.816	< 0.001
*hya3*	0.1281	0.0697	6.832	0.009
*hya4*	0.1983	0.1432	4.390	0.036

*Note:* Hematophagous leeches were designated as foreground branches, whereas non‐hematophagous species together with outgroup taxa were treated as background branches. *hya5* was excluded from branch‐model comparisons because orthologous sequences were absent in non‐hematophagous species.

Importantly, all estimated *ω* values were substantially lower than 1, indicating that purifying selection remains the primary force driving the evolution of the hyaluronidase gene family.

### 3.4 Comparative Expression of Hyaluronidase Genes in Leeches With Different Feeding Strategies

**FIGURE 2:**
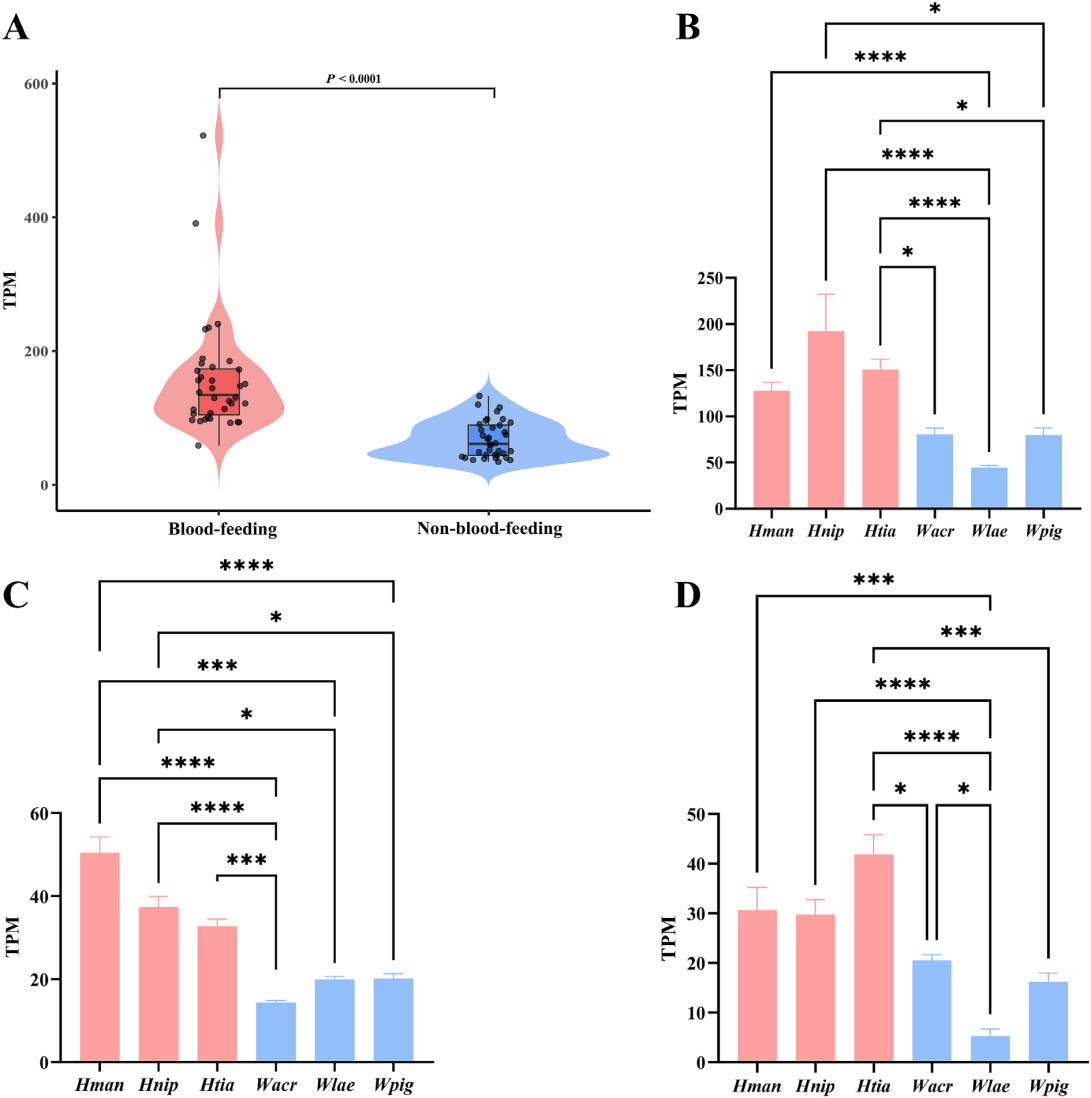
Comparative expression of hyaluronidase genes. (A) Comparison of total hyaluronidase expression between hematophagous and non‐hematophagous leeches; (B) Comparison of total hyaluronidase expression among leech species; (C) Expression of *hya1*; (D) Expression of *hya2*. Red and blue bars represent hematophagous and non‐hematophagous leeches, respectively. Error bars indicate mean ± SEM *p < 0.05, ***p < 0.001, ****p < 0.0001; comparisons without asterisks are not significant.

To evaluate the association between hyaluronidase gene expression and feeding strategy in leeches, the six species were grouped into hematophagous and non‐hematophagous categories, and transcriptomic TPM data were analyzed. The results showed that the overall expression levels of hyaluronidase genes were significantly higher in hematophagous leeches than in non‐hematophagous species (Mann–Whitney *U* test, *Z* = −6.318, *p* < 0.0001; Figure 2).

To further account for phylogenetic non‐independence among species, we additionally performed phylogenetic ANOVA (Table S3 in Supporting Information S2). Phylogenetic ANOVA revealed that total expression of all five hyaluronidase genes was also significantly higher in hematophagous leeches (*F* = 16.745, *p* = 0.015), with *hya1*, *hya2*, and *hya5* showing significant differences individually (*p* < 0.05). Thus, despite the conservative nature of phylogenetic corrections, the direction and significance of the expression differences remain consistent with the non‐parametric results, supporting the conclusion that hematophagous leeches have elevated hyaluronidase expression.

**FIGURE 3:**
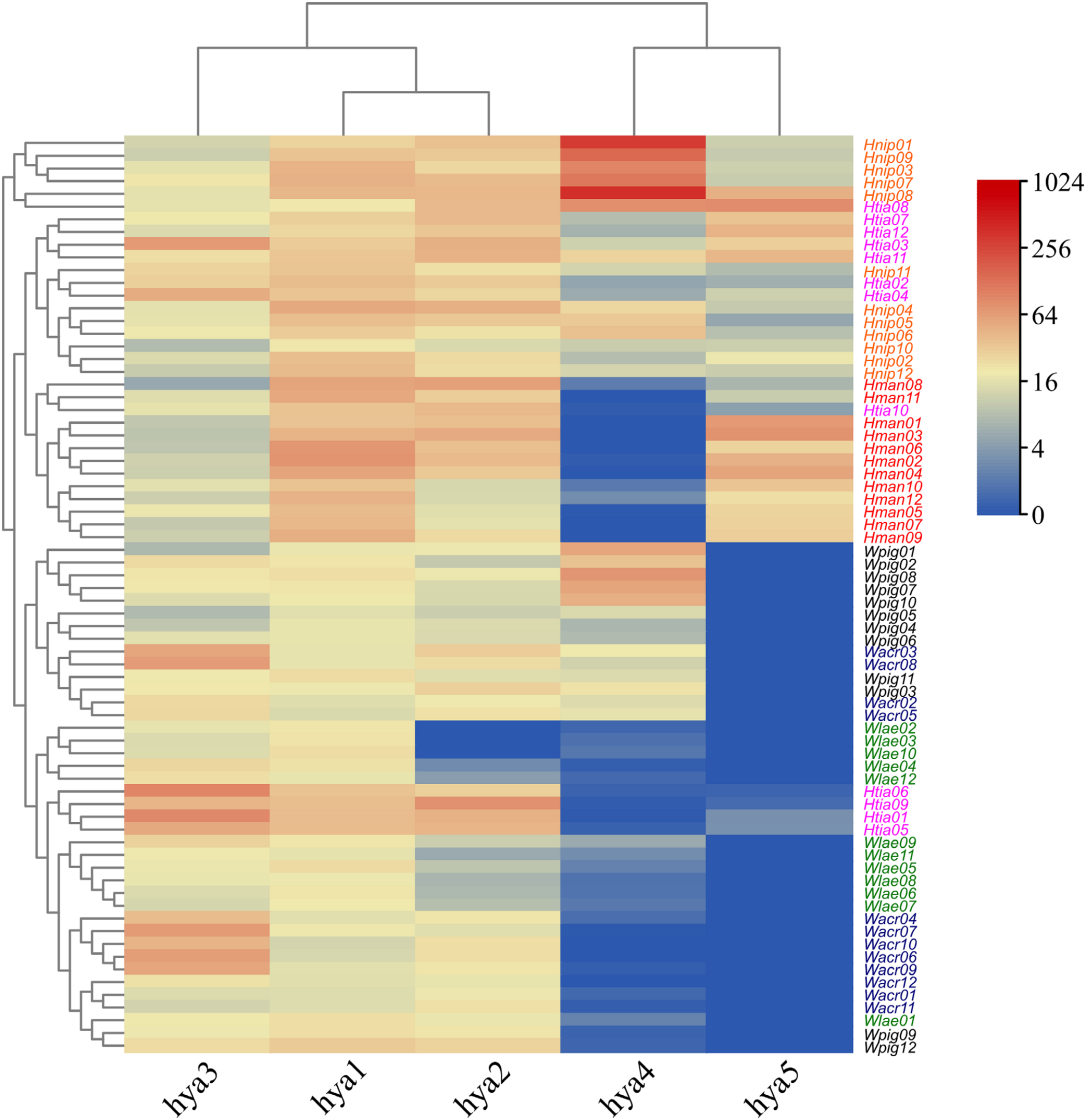
Expression heatmap of the hyaluronidase gene family across leech species. The color of sample labels indicates the species: *H. manillensis* (red), *H. nipponia* (orange), *H. tianjinensis* (purple), *W. laevis* (green), *W. acranulata* (blue), *W. pigra* (black). Expression levels are shown according to the color scale, with red representing high expression and blue representing low expression.

Among individual hyaluronidase gene family members, the expression patterns of *hya1* and *hya2* were broadly consistent with phylogenetic relationships, with hematophagous species generally exhibiting higher expression levels than non‐hematophagous species. Although other hyaluronidase genes also showed interspecific variation in expression, their expression patterns were less clearly associated with phylogenetic relationships compared with those of *hya1* and *hya2* (Figure 3, Figure S1 in Supporting Information S2).

**FIGURE 4:**
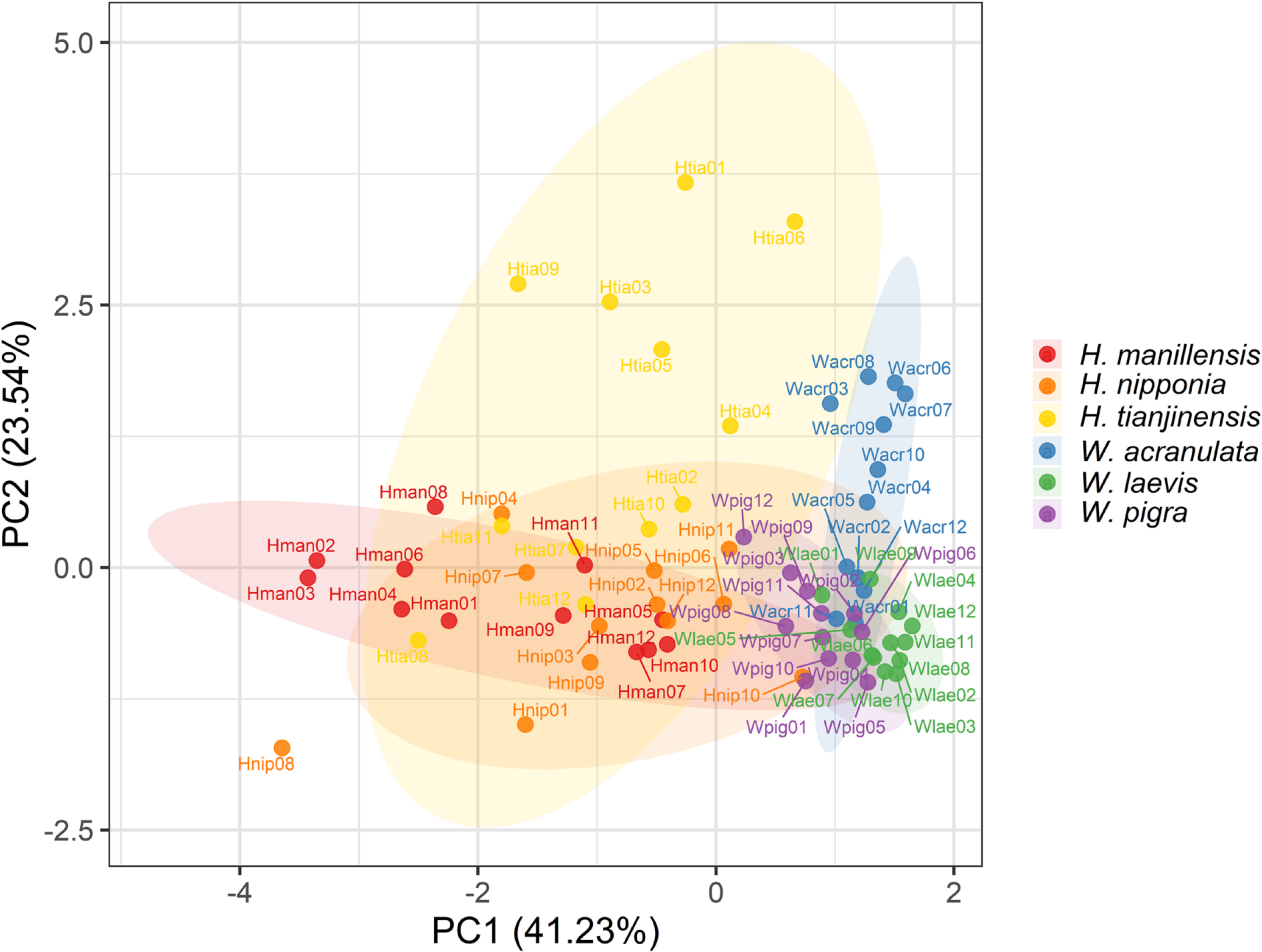
PCA of hyaluronidase gene family expression patterns across different leech species. Each point represents an individual sample, color‐coded by species: Red/orange/yellow for hematophagous species ( *H. manillensis* , *H. nipponia*, *H. tianjinensis*) and blue/green/purple for non‐hematophagous species (*W. acranulata*, *W. laevis*, *W. pigra*). Ellipses, shaded in red for hematophagous and blue for non‐hematophagous species, indicate 95% confidence intervals for each feeding type, highlighting feeding‐type‐specific expression patterns. The first two principal components (PC1 and PC2) explain 41.23% and 23.54% of the total variance, respectively.

Hierarchical clustering analysis and principal component analysis (PCA) further revealed a clear separation between hematophagous and non‐hematophagous species in the overall expression profiles of the hyaluronidase gene family (Figures 3 and 4). Hematophagous species clustered together and were characterized by high expression levels across multiple hyaluronidase gene family members, whereas non‐hematophagous species formed a separate cluster with generally lower expression levels or with some genes being barely detectable. Compared with other species, *H. tianjinensis* samples showed relatively greater dispersion in the PCA space, which may reflect transcriptomic heterogeneity among biological replicates within this species. These patterns indicate that variation in hyaluronidase gene family expression is strongly associated with feeding strategy.

Overall, hyaluronidase genes exhibited consistently higher expression in hematophagous leeches, and the observed expression differences were closely related to both species divergence and feeding strategy. This pattern suggests that hyaluronidase may undergo functional enhancement through transcriptional regulation during the adaptation to hematophagy. Together with the results from gene copy retention and positive selection analyses, these findings further support a key role for hyaluronidase in the adaptive evolution of hematophagous leeches.

### 3.5 Expression Divergence and Regulatory Patterns of the Hyaluronidase Gene Family

**FIGURE 5:**
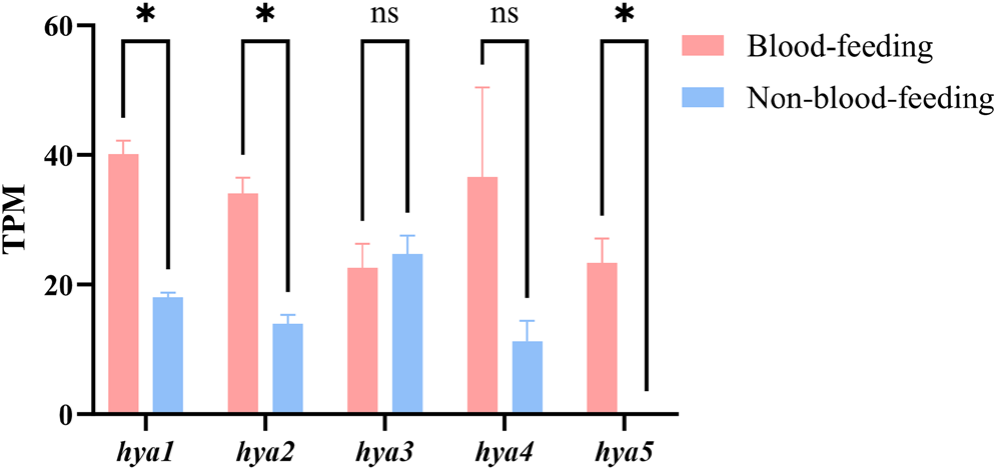
Comparative expression of individual hyaluronidase gene family members between hematophagous and non‐hematophagous leeches. Error bars indicate mean ± SEM *p < 0.05; ns, not significant.

**FIGURE 6:**
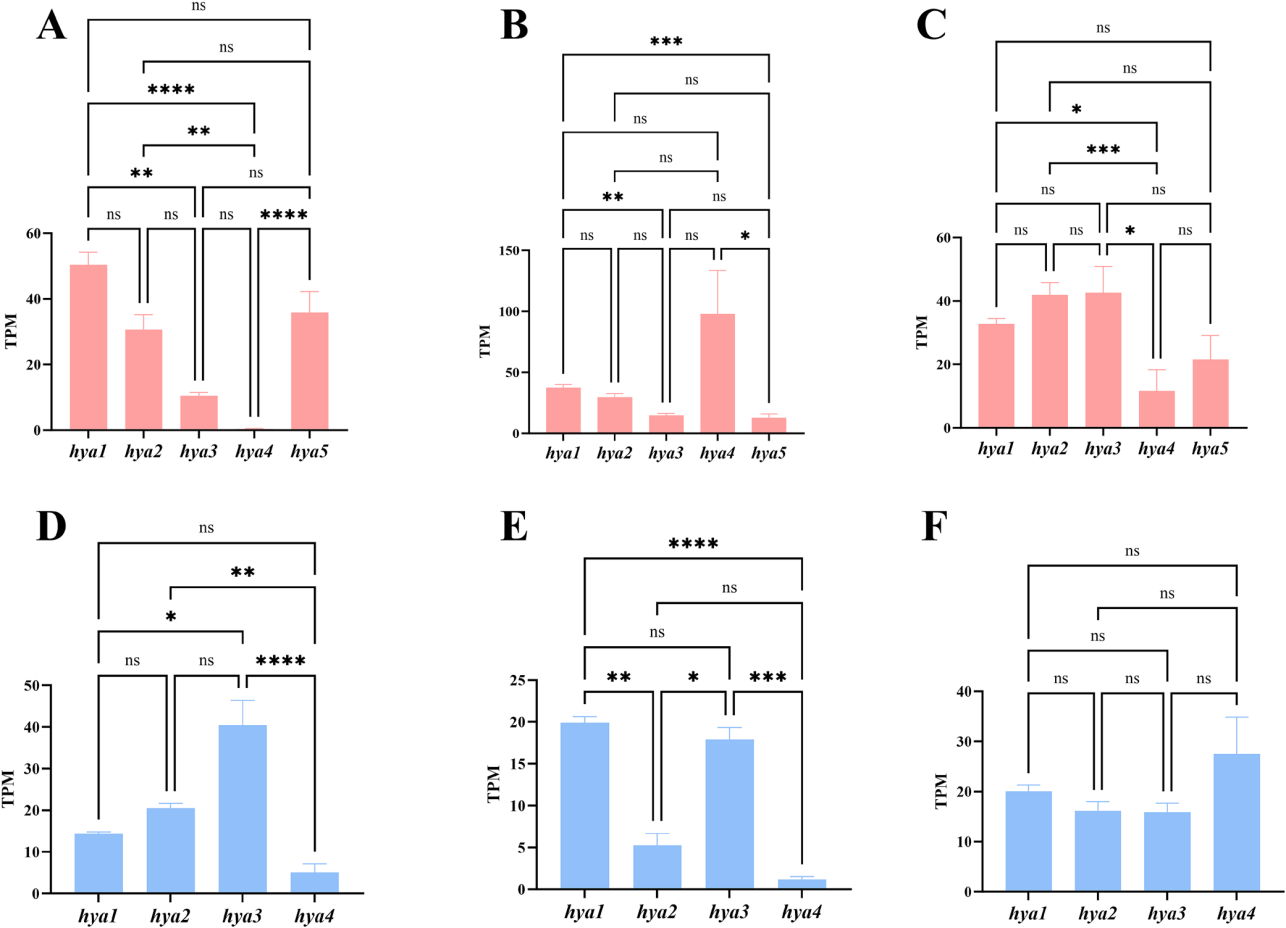
Expression levels of individual hyaluronidase gene family members within each leech species. (A) *H. manillensis* ; (B) *H. nipponia*; (C) *H. tianjinensis*; (D) *W. acranulata*; (E) *W. laevis*; (F) *W. pigra*. Red and blue bars represent hematophagous and non‐hematophagous leeches, respectively. Error bars represent mean ± SEM *p < 0.05, **p < 0.01, ***p < 0.001, ****p < 0.0001; ns, not significant.

To further investigate the expression regulation of hyaluronidase gene family members, we compared the total expression of each member, the expression of each member between hematophagous and non‐hematophagous leeches, and the expression levels of different members within each species. The results revealed significant differences among gene members (*H* = 55.01, df = 4, *p* < 0.0001), with *hya1* and *hya2* showing significantly higher expression than most other members. Furthermore, the expression of *hya1* and *hya2* was significantly higher in hematophagous leeches than in non‐hematophagous species (Figure 5), highlighting their key roles in hyaluronidase function. Within‐species analyses further indicated functional divergence among members (Table 3, Figure 6), with significant expression differences observed in all species except *W. pigra*. Hematophagous leeches maintained expression of multiple members, whereas *hya5* was absent in non‐hematophagous species.

**TABLE 3:** Statistical results of expression differences among hyaluronidase gene family members within each leech species.

Species	*χ* ^2^	df	*p*
*H. manillensis*	37.27	4	< 0.001
*H. nipponia*	25.67	4	< 0.001
*H. tianjinensis*	16.53	4	< 0.001
*W. acranulata*	26.30	3	< 0.001
*W. laevis*	30.10	3	< 0.001
*W. pigra*	5.30	3	0.151

Taken together, these results indicate pronounced divergence in the expression regulation of the hyaluronidase gene family. The consistently high expression of *hya1 and hya2*, together with the lineage‐specific presence of *hya5*, likely underlies the enhanced hyaluronan‐degrading capacity of hematophagous leeches and reflects functional optimization and adaptive regulatory modulation of this gene family during the evolution of hematophagy. Phylogenetic ANOVA further confirmed the significantly higher expression of *hya1, hya2*, and *hya5* in hematophagous leeches (see Table S3).

## 4 Discussion

### 4.1 Evolutionary Retention of Ancestral Hyaluronidase Gene Copies

Among the leech species examined in this study, hematophagous taxa (*Hirudo* and *Hirudinaria*) possess a greater number of retained hyaluronidase gene family members than the non‐hematophagous genus *Whitmania*. Phylogenetic analyses showed that each hyaluronidase gene family member (*hya1*–*hya5*) formed a well‐supported monophyletic clade, indicating that divergence among ancestral paralogous copies occurred prior to species differentiation. Therefore, the observed differences in gene copy number between hematophagous and non‐hematophagous leeches are more likely attributable to differential retention of ancestral hyaluronidase gene copies rather than lineage‐specific gene duplication events occurring after the evolution of hematophagy.

The long‐term divergence in feeding strategy may underlie this differential retention. Hematophagous leeches have persistently relied on physiological processes such as tissue penetration, degradation of the host ECM, and regulation of anticoagulation, making it advantageous to retain multiple ancestral gene family members and maintain their expression. In contrast, non‐hematophagous leeches do not depend on blood‐feeding–related physiological functions and primarily obtain nutrition through predation on invertebrates, although dietary preferences vary among species. For example, within the genus *Whitmania*, *W. pigra* is a typical macrophagous predator mainly feeding on mollusks, *W. laevis* has a broader diet including mollusks and aquatic insect larvae, and *W. acranulata* primarily consumes aquatic oligochaetes and insect larvae (Zhao et al. 2025; Liu et al. 2024). In these non‐hematophagous species, hyaluronidase gene family members are more prone to loss or functional degradation under the birth–death model of multigene family evolution, such that long‐term retention depends on ecological context and functional demand (Nei and Rooney 2005).

Functionally, a greater number of retained hyaluronidase gene family members may confer potential advantages through two non‐mutually exclusive mechanisms. First, a dosage effect, whereby multiple ancestral gene copies increase overall expression levels, thereby enhancing degradation of host ECM components such as hyaluronan and facilitating tissue penetration and dispersal. Second, different gene family members may gradually diverge in subfunctional roles, potentially improving hematophagic efficiency. Although direct experimental validation of functional differences among hyaluronidase gene family members is currently lacking, similar evolutionary patterns have been widely documented in other hematophagous taxa. For example, in ticks, gene families encoding lipocalins, Kunitz‐type protease inhibitors, and serpins have undergone extensive duplication and diversification, producing numerous paralogs that contribute to anticoagulation, immune modulation, and suppression of host inflammatory responses, thereby underpinning blood‐feeding capability (Mans et al. 2017). This analogy suggests that, in hematophagous lineages, retention of a greater number of ancestral gene family members associated with tissue degradation or host modulation may play an important role in blood‐feeding behavior.

### 4.2 Selective Pressure Characteristics of Hyaluronidase Genes

Although the retention of ancestral copies provides a potential basis for the adaptive evolution of hyaluronidase genes, these genes remain strongly constrained at key functional sites, resulting in overall high sequence conservation. Our analyses show that the overall *ω* (*dN*/*dS*) values of the hyaluronidase gene family are well below 1, indicating that purifying selection is the predominant force shaping the evolution of this gene family. This pattern is consistent with the biochemical properties of the enzyme, as hyaluronidases must preserve highly conserved catalytic core domains to ensure that their ability to cleave *β*‐1,3‐glucuronide bonds of hyaluronan is not compromised by deleterious mutations (Lu et al. 2025).

Nevertheless, the consistently higher *ω* values observed in hematophagous lineages suggest that their selective constraint patterns differ from those of non‐hematophagous species (*p* < 0.001). Because all estimated *ω* values remained below 1, this pattern is more consistent with relaxed purifying selection rather than classical positive selection. This finding suggests that, while maintaining core enzymatic functions, hyaluronidase genes in hematophagous lineages may tolerate a certain degree of sequence variation, potentially conferring relatively greater evolutionary flexibility.

Notably, selective pressure differences among hyaluronidase paralogs were not uniform. Significant differences between hematophagous and non‐hematophagous lineages were detected for *hya2*–*hya4*, whereas no significant divergence was observed for *hya1* (Table 2). This heterogeneity suggests that different hyaluronidase paralogs may have experienced distinct changes in selective constraints following gene duplication, which may be associated with functional divergence trends among gene family members.

Overall, the evolutionary pattern of the hyaluronidase gene family in the leech species examined in this study reflects both functional conservation and evolutionary flexibility. On the one hand, strong purifying selection has maintained the stability of essential enzymatic functions; on the other hand, relatively relaxed selective constraints in hematophagous lineages may have promoted sequence diversification during long‐term adaptation to a blood‐feeding lifestyle.

### 4.3 Expression of Hyaluronidase and Adaptation to Blood‐Feeding Behavior

Hyaluronidase genes exhibit not only adaptive evolution at the sequence level but also transcriptional regulation patterns consistent with feeding strategies. During feeding, hyaluronidase functions as a “spreading factor,” with its efficacy highly dependent on local enzyme concentration (Claude 1937). In the temporally constrained blood‐feeding process, leeches must rapidly disrupt host skin and ECM and facilitate the diffusion of anticoagulant, analgesic, and other bioactive molecules. High expression of hyaluronidase genes ensures sufficient enzyme availability within a short time frame, enabling prompt ECM degradation and blood flow, thereby providing essential conditions for successful feeding.

Our expression analyses indicate that the total hyaluronidase expression in hematophagous leeches is significantly higher than in non‐hematophagous species (*p* < 0.0001). This pattern remains consistent after accounting for phylogenetic non‐independence using phylogenetic ANOVA. Within hematophagous species, core gene family members *hya1* and *hya2* consistently exhibit high expression, suggesting that they may play fundamental roles in maintaining feeding efficiency. Similar trends are observed for other blood‐feeding–related genes; for instance, *LDTI* shows markedly higher expression in hematophagous leeches, with *H. manillensis* displaying the highest expression and strongest anticoagulant activity (Xiao et al. 2025).

By contrast, non‐hematophagous leeches exhibit generally lower hyaluronidase expression, accompanied by the loss of specific gene family members such as *hya5*. This pattern aligns with their altered feeding strategies: lacking reliance on rapid host tissue degradation, the demand for high hyaluronidase dosage decreases, leading to a reduction in both the composition of gene family members and their expression levels. Furthermore, among non‐hematophagous species, *W. laevis* exhibits lower total hyaluronidase expression than *W. pigra*, consistent with previous reports that *W. laevis* lacks detectable jaw plates and shows more complete regression of blood‐feeding traits (Qiao et al. 2013). These findings indicate that the expression regulation of key effector proteins such as hyaluronidase is closely linked to feeding strategy in the leech species examined here, although the causal relationship with blood‐feeding behavior remains to be experimentally validated.

Accordingly, the markedly elevated expression of the hyaluronidase gene family in hematophagous leeches likely contributes to blood‐feeding behavior and is associated with feeding strategy.

It should be noted that the number of independent evolutionary contrasts in feeding strategy in the present dataset is very limited, which may reduce statistical power. In addition, differences in effective population size (*N*_e_) among major clades may influence variation in *dN*/*dS* among lineages. Because independent estimates of *N*_e_ are not available, we cannot disentangle the relative contributions of feeding strategy and demographic history. Furthermore, all samples were collected from a single geographic location, which may not fully capture intraspecific variation. Collectively, these limitations may affect the strength of the inference but do not alter the overall pattern observed.

### 4.4 Lineage‐Specific Variation in *hya4* Expression

Although hematophagous leeches generally exhibit multi‐copy retention and high expression of the hyaluronidase gene family, this pattern is not completely uniform across all species. In this study, *hya4* showed lower expression in *H. tianjinensis* compared with the closely related *H. nipponia*, which exhibits relatively higher expression levels.

This observation suggests that, even within a relatively conserved gene family, individual paralogous copies may exhibit lineage‐specific differences in expression levels, leading to variation in gene family expression profiles among closely related hematophagous leeches. Such differences are more likely to reflect regulatory divergence rather than structural gene loss. Notably, similar expression divergence has been reported for other blood‐feeding–related effector genes. For example, higher hirudin expression has been observed in *H. nipponia* compared with *H. tianjinensis* (Yin et al. 2025), suggesting that these closely related species may differ in the transcriptional regulation of key genes associated with hematophagy. Overall, the observed variation in *hya4* expression reflects lineage‐specific transcriptional divergence within the hyaluronidase gene family in medicinal leeches, highlighting regulatory differences among closely related hematophagous species. These differences may also have descriptive relevance in the context of medicinal leech research and species characterization. The Pharmacopeia of the People's Republic of China currently lists *H. nipponia* as a standard medicinal leech species, whereas *H. tianjinensis* has not been included in mainstream medicinal usage (Chinese Pharmacopoeia Commission 2020). However, it should be emphasized that this study does not directly assess pharmacological activity; therefore, no conclusions can be drawn regarding potential differences in medicinal efficacy between species.

Taken together, although this study provides a comparative evolutionary and transcriptomic framework for understanding the hyaluronidase gene family, several limitations should be acknowledged. Functional interpretations are primarily based on indirect evidence from sequence evolution and transcriptomic data, without direct experimental validation at the protein or cellular level. In addition, transcript abundance may not fully reflect protein levels due to post‐transcriptional regulation. Therefore, our conclusions should be interpreted within the context of comparative evolutionary genomics and gene expression analyses. Future studies integrating proteomic data and functional assays will help to further validate these findings.

## 5 Conclusions

This study systematically analyzed the hyaluronidase gene family in leeches with different feeding strategies, examining gene copy number, selective pressures, and expression patterns, thereby revealing multiple evolutionary patterns associated with hematophagy. Our results show that each hyaluronidase gene family member forms a well‐supported monophyletic group, and these genes are highly conserved across species, indicating that these genes diverged prior to species differentiation. Hematophagous leeches have not gained additional hyaluronidase copies through recent gene duplication; instead, the long‐term retention of ancestral multi‐copy genes may contribute to the maintenance of ECM degradation processes relevant to blood‐feeding. At the molecular evolutionary level, the hyaluronidase gene family is predominantly shaped by purifying selection (*ω* < 1), reflecting strong functional constraint on its essential enzymatic roles. However, hematophagous lineages exhibit relatively elevated *ω* values, suggesting a moderate relaxation of selective constraints rather than strong positive selection, indicating limited but potentially functional evolutionary flexibility in blood‐feeding species. Expression analyses further demonstrated that hyaluronidase genes were consistently more highly expressed in hematophagous leeches, with *hya1* and *hya2* showing particularly elevated expression levels, suggesting that transcriptional regulation may play an important role in the association between hyaluronidase function and blood‐feeding strategy. Notably, variation in *hya4* expression in *H. tianjinensis* suggests that individual gene copies may exhibit lineage‐specific differences in expression among closely related species.

Overall, the evolution of the hyaluronidase gene family is shaped by the combined effects of gene retention, sequence‐level constraint, and expression regulation in the Arhynchobdellida leech species examined in this study. Whether comparable patterns of gene retention, selective constraint, and expression regulation occur in other leech lineages, including the order Rhynchobdellida, remains to be tested in future studies.

## Author Contributions

**Rujiao Sun:** formal analysis (equal), investigation (equal), methodology (lead), visualization (lead), writing – original draft (lead), writing – review and editing (equal). **Xingke Fu:** data curation (equal), investigation (equal), methodology (equal), visualization (equal). **Rui Ai:** data curation (equal), formal analysis (equal), visualization (equal). **Zuhao Huang:** funding acquisition (equal), project administration (equal). **Huanhuan Li:** data curation (equal), formal analysis (equal). **Lizhou Tang:** investigation (equal), resources (equal). **Huanhuan Chen:** investigation (equal), resources (equal). **Xiongsheng Zhang:** investigation (equal), resources (equal). **Fang Zhao:** conceptualization (lead), methodology (lead), supervision (lead), writing – review and editing (equal). **Gonghua Lin:** conceptualization (equal), funding acquisition (lead), project administration (equal), resources (lead), writing – review and editing (lead).

## Funding

This research was funded by the National Natural Science Foundation of China (grant number 82260742); the Foundation of Yunnan International Joint Laboratory with South and Southeast Asia for the Integrated Development of Animal‐Derived Anti‐Thrombosis Chinese Medicine (grant number 202503AP140025); and the Foundation of Key Laboratory of Jiangxi Province for Biological Invasion and Biosecurity (grant number 2023SSY02111).

## Conflicts of Interest

The authors declare no conflicts of interest.

## Supporting information


**Supporting Information S1:** FASTA file of hyaluronidase gene family CDSs from eight leech species and one outgroup.
**Supporting Information S2:** Table S1 (pairwise sequence identities of hyaluronidase gene family members in eight leech species), Table S2 (summary of RNA‐seq read mapping rates for all samples), Table S3 (phylogenetic ANOVA comparing hyaluronidase gene expression between hematophagous and non‐hematophagous leeches) and Figure S1 (comparative expression of hyaluronidase gene family members *hya3*–*hya5* across leech species).
**Supporting Information S3:** Excel file of TPM values for hyaluronidase gene family members (*hya1*–*hya5*) in six leech species, 12 samples per species.

## Data Availability

Raw RNA‐seq data used in this study have been deposited in the China National GeneBank DataBase (CNGBdb), which is maintained by the National Genomics Data Center (NGDC), under two BioProject accession numbers: PRJCA046291 (https://ngdc.cncb.ac.cn/bioproject/browse/PRJCA046291) and PRJCA058083 (https://ngdc.cncb.ac.cn/bioproject/browse/PRJCA058083). All other data supporting the findings of this study are available within the article and its Supporting Information.
