# Supplementary material for: Feeding Strategy Shapes the Evolution of the Hyaluronidase Gene Family in Medicinal Leeches
Source: Ecol Evol. 2026 Jul 14;16(7):e74049. doi: 10.1002/ece3.74049 (PMC13368403; doi:10.1002/ece3.74049)
Supplement: Supplementary file 1 — Supporting Information S1: FASTA file of hyaluronidase gene family CDSs from eight leech species and one outgroup. Supporting Information S2: Table S1 (pairwise sequence identities of hyaluronidase gene family members in eight leech species), Table S2 (summary of RNA‐seq read mapping rates for all samples), Table S3 (phylogenetic ANOVA comparing hyaluronidase gene expression between hematophagous and non‐hematophagous leeches) and Figure S1 (comparative expression of hyaluronidase gene family members hya3–hya5 across leech species). Supporting Information S3: Excel file of TPM values for hyaluronidase gene family members (hya1–hya5) in six leech species, 12 samples per species. [file ECE3-16-e74049-s001.zip › ece374049-sup-0002-Supinfo02.docx]

Supplementary Material S2

This file includes the following supplementary items:

Supplementary Table S1. Pairwise sequence identities of hyaluronidase gene family members in eight leech species.

Supplementary Table S2. Summary of RNA-seq read mapping rates for all samples.

Supplementary Table S3. Phylogenetic ANOVA comparing hyaluronidase gene expression between hematophagous and non-hematophagous leeches.

Supplementary Figure S1. Comparative expression of hyaluronidase gene family members *hya3*–*hya5* across leech species.

**Supplementary Table S1.** Pairwise sequence identities of hyaluronidase gene family members in eight leech species.

| Gene1 | Gene2 | Pairwise sequence identity (%) |
| --- | --- | --- |
| *hya1-Hman* | *hya1-Hbpl* | 97.28 |
| *hya1-Hman* | *hya1-Hmed* | 84.82 |
| *hya1-Hman* | *hya1-Hnip* | 84.43 |
| *hya1-Hman* | *hya1-Htia* | 83.90 |
| *hya1-Hman* | *hya1-Wacr* | 82.82 |
| *hya1-Hman* | *hya1-Wlae* | 83.04 |
| *hya1-Hman* | *hya1-Wpig* | 83.70 |
| *hya1-Hbpl* | *hya1-Hmed* | 84.49 |
| *hya1-Hbpl* | *hya1-Hnip* | 84.49 |
| *hya1-Hbpl* | *hya1-Htia* | 83.70 |
| *hya1-Hbpl* | *hya1-Wacr* | 83.09 |
| *hya1-Hbpl* | *hya1-Wlae* | 83.17 |
| *hya1-Hbpl* | *hya1-Wpig* | 83.70 |
| *hya1-Hmed* | *hya1-Hnip* | 91.91 |
| *hya1-Hmed* | *hya1-Htia* | 91.78 |
| *hya1-Hmed* | *hya1-Wacr* | 90.46 |
| *hya1-Hmed* | *hya1-Wlae* | 91.58 |
| *hya1-Hmed* | *hya1-Wpig* | 91.78 |
| *hya1-Hnip* | *hya1-Htia* | 97.44 |
| *hya1-Hnip* | *hya1-Wacr* | 92.89 |
| *hya1-Hnip* | *hya1-Wlae* | 94.67 |
| *hya1-Hnip* | *hya1-Wpig* | 95.00 |
| *hya1-Htia* | *hya1-Wacr* | 92.89 |
| *hya1-Htia* | *hya1-Wlae* | 94.94 |
| *hya1-Htia* | *hya1-Wpig* | 95.40 |
| *hya1-Wacr* | *hya1-Wlae* | 92.63 |
| *hya1-Wacr* | *hya1-Wpig* | 92.24 |
| *hya1-Wlae* | *hya1-Wpig* | 97.17 |
| *hya2-Hman* | *hya2-Hbpl* | 97.02 |
| *hya2-Hman* | *hya2-Hmed* | 85.39 |
| *hya2-Hman* | *hya2-Hnip* | 84.96 |
| *hya2-Hman* | *hya2-Htia* | 85.16 |
| *hya2-Hman* | *hya2-Wacr* | 84.36 |
| *hya2-Hman* | *hya2-Wlae* | 85.29 |
| *hya2-Hman* | *hya2-Wpig* | 85.55 |
| *hya2-Hbpl* | *hya2-Hmed* | 85.66 |
| *hya2-Hbpl* | *hya2-Hnip* | 84.96 |
| *hya2-Hbpl* | *hya2-Htia* | 85.22 |
| *hya2-Hbpl* | *hya2-Wacr* | 84.23 |
| *hya2-Hbpl* | *hya2-Wlae* | 85.35 |
| *hya2-Hbpl* | *hya2-Wpig* | 85.49 |
| *hya2-Hmed* | *hya2-Hnip* | 91.37 |
| *hya2-Hmed* | *hya2-Htia* | 91.77 |
| *hya2-Hmed* | *hya2-Wacr* | 90.24 |
| *hya2-Hmed* | *hya2-Wlae* | 91.77 |
| *hya2-Hmed* | *hya2-Wpig* | 91.90 |
| *hya2-Hnip* | *hya2-Htia* | 97.95 |
| *hya2-Hnip* | *hya2-Wacr* | 94.17 |
| *hya2-Hnip* | *hya2-Wlae* | 96.16 |
| *hya2-Hnip* | *hya2-Wpig* | 96.36 |
| *hya2-Htia* | *hya2-Wacr* | 94.57 |
| *hya2-Htia* | *hya2-Wlae* | 96.42 |
| *hya2-Htia* | *hya2-Wpig* | 96.62 |
| *hya2-Wacr* | *hya2-Wlae* | 94.30 |
| *hya2-Wacr* | *hya2-Wpig* | 94.43 |
| *hya2-Wlae* | *hya2-Wpig* | 99.01 |
| *hya3-Hman* | *hya3-Hbpl* | 96.64 |
| *hya3-Hman* | *hya3-Hmed* | 82.75 |
| *hya3-Hman* | *hya3-Hnip* | 81.82 |
| *hya3-Hman* | *hya3-Htia* | 82.44 |
| *hya3-Hman* | *hya3-Wacr* | 81.24 |
| *hya3-Hman* | *hya3-Wlae* | 82.17 |
| *hya3-Hman* | *hya3-Wpig* | 81.76 |
| *hya3-Hbpl* | *hya3-Hmed* | 83.51 |
| *hya3-Hbpl* | *hya3-Hnip* | 82.03 |
| *hya3-Hbpl* | *hya3-Htia* | 82.58 |
| *hya3-Hbpl* | *hya3-Wacr* | 81.72 |
| *hya3-Hbpl* | *hya3-Wlae* | 82.44 |
| *hya3-Hbpl* | *hya3-Wpig* | 81.89 |
| *hya3-Hmed* | *hya3-Hnip* | 90.52 |
| *hya3-Hmed* | *hya3-Htia* | 90.93 |
| *hya3-Hmed* | *hya3-Wacr* | 89.67 |
| *hya3-Hmed* | *hya3-Wlae* | 90.65 |
| *hya3-Hmed* | *hya3-Wpig* | 90.65 |
| *hya3-Hnip* | *hya3-Htia* | 97.12 |
| *hya3-Hnip* | *hya3-Wacr* | 92.16 |
| *hya3-Hnip* | *hya3-Wlae* | 95.27 |
| *hya3-Hnip* | *hya3-Wpig* | 95.13 |
| *hya3-Htia* | *hya3-Wacr* | 92.65 |
| *hya3-Htia* | *hya3-Wlae* | 95.54 |
| *hya3-Htia* | *hya3-Wpig* | 95.27 |
| *hya3-Wacr* | *hya3-Wlae* | 92.51 |
| *hya3-Wacr* | *hya3-Wpig* | 92.37 |
| *hya3-Wlae* | *hya3-Wpig* | 97.33 |
| *hya4-Hman* | *hya4-Hbpl* | 87.67 |
| *hya4-Hman* | *hya4-Hmed* | 73.15 |
| *hya4-Hman* | *hya4-Hnip* | 72.46 |
| *hya4-Hman* | *hya4-Htia* | 72.39 |
| *hya4-Hman* | *hya4-Wacr* | 72.12 |
| *hya4-Hman* | *hya4-Wlae* | 72.65 |
| *hya4-Hman* | *hya4-Wpig* | 71.92 |
| *hya4-Hbpl* | *hya4-Hmed* | 73.55 |
| *hya4-Hbpl* | *hya4-Hnip* | 73.00 |
| *hya4-Hbpl* | *hya4-Htia* | 73.20 |
| *hya4-Hbpl* | *hya4-Wacr* | 72.40 |
| *hya4-Hbpl* | *hya4-Wlae* | 72.73 |
| *hya4-Hbpl* | *hya4-Wpig* | 72.53 |
| *hya4-Hmed* | *hya4-Hnip* | 87.38 |
| *hya4-Hmed* | *hya4-Htia* | 87.58 |
| *hya4-Hmed* | *hya4-Wacr* | 87.12 |
| *hya4-Hmed* | *hya4-Wlae* | 88.45 |
| *hya4-Hmed* | *hya4-Wpig* | 88.51 |
| *hya4-Hnip* | *hya4-Htia* | 95.70 |
| *hya4-Hnip* | *hya4-Wacr* | 92.72 |
| *hya4-Hnip* | *hya4-Wlae* | 93.45 |
| *hya4-Hnip* | *hya4-Wpig* | 93.92 |
| *hya4-Htia* | *hya4-Wacr* | 92.39 |
| *hya4-Htia* | *hya4-Wlae* | 93.72 |
| *hya4-Htia* | *hya4-Wpig* | 94.11 |
| *hya4-Wacr* | *hya4-Wlae* | 92.99 |
| *hya4-Wacr* | *hya4-Wpig* | 93.39 |
| *hya4-Wlae* | *hya4-Wpig* | 98.28 |
| *hya5-Hman* | *hya5-Hbpl* | 95.62 |
| *hya5-Hman* | *hya5-Hmed* | 81.20 |
| *hya5-Hman* | *hya5-Hnip* | 79.96 |
| *hya5-Hman* | *hya5-Htia* | 80.03 |
| *hya5-Hbpl* | *hya5-Hmed* | 81.47 |
| *hya5-Hbpl* | *hya5-Hnip* | 80.03 |
| *hya5-Hbpl* | *hya5-Htia* | 80.03 |
| *hya5-Hmed* | *hya5-Hnip* | 87.36 |
| *hya5-Hmed* | *hya5-Htia* | 87.62 |
| *hya5-Hnip* | *hya5-Htia* | 94.17 |

**Supplementary Table S2.** Summary of RNA-seq read mapping rates for all samples.

| Sample | Mapping rate (%) |
| --- | --- |
| Hman01 | 44.23 |
| Hman02 | 46.31 |
| Hman03 | 42.25 |
| Hman04 | 40.88 |
| Hman05 | 41.16 |
| Hman06 | 44.18 |
| Hman07 | 38.39 |
| Hman08 | 43.86 |
| Hman09 | 40.92 |
| Hman10 | 39.16 |
| Hman11 | 41.85 |
| Hman12 | 41.52 |
| Hnip01 | 38.39 |
| Hnip02 | 41.61 |
| Hnip03 | 46.60 |
| Hnip04 | 39.11 |
| Hnip05 | 38.58 |
| Hnip06 | 45.64 |
| Hnip07 | 43.39 |
| Hnip08 | 42.25 |
| Hnip09 | 43.47 |
| Hnip10 | 50.33 |
| Hnip11 | 42.54 |
| Hnip12 | 43.21 |
| Htia01 | 43.89 |
| Htia02 | 44.87 |
| Htia03 | 42.73 |
| Htia04 | 47.75 |
| Htia05 | 47.65 |
| Htia06 | 45.81 |
| Htia07 | 42.46 |
| Htia08 | 45.44 |
| Htia09 | 44.98 |
| Htia10 | 47.80 |
| Htia11 | 44.11 |
| Htia12 | 45.91 |
| Wacr01 | 49.45 |
| Wacr02 | 51.78 |
| Wacr03 | 54.05 |
| Wacr04 | 54.26 |
| Wacr05 | 51.62 |
| Wacr06 | 58.50 |
| Wacr07 | 52.70 |
| Wacr08 | 46.82 |
| Wacr09 | 55.68 |
| Wacr10 | 53.31 |
| Wacr11 | 55.02 |
| Wacr12 | 51.62 |
| Wlae01 | 45.05 |
| Wlae02 | 43.39 |
| Wlae03 | 43.40 |
| Wlae04 | 45.14 |
| Wlae05 | 44.60 |
| Wlae06 | 45.33 |
| Wlae07 | 45.21 |
| Wlae08 | 43.18 |
| Wlae09 | 43.78 |
| Wlae10 | 43.37 |
| Wlae11 | 45.28 |
| Wlae12 | 43.17 |
| Wpig01 | 53.70 |
| Wpig02 | 53.43 |
| Wpig03 | 57.16 |
| Wpig04 | 52.86 |
| Wpig05 | 50.74 |
| Wpig06 | 56.16 |
| Wpig07 | 51.52 |
| Wpig08 | 64.41 |
| Wpig09 | 64.97 |
| Wpig10 | 60.78 |
| Wpig11 | 50.48 |
| Wpig12 | 48.27 |

**Supplementary Table S3.** Phylogenetic ANOVA comparing hyaluronidase gene expression between hematophagous and non-hematophagous leeches.

| Trait | F value | P value | Significance |
| --- | --- | --- | --- |
| *hya1* expression | 15.706 | 0.021 | * |
| *hya2* expression | 11.170 | 0.049 | * |
| *hya3* expression | 0.024 | 0.899 | ns |
| *hya4* expression | 0.730 | 0.517 | ns |
| *hya5* expression | 12.274 | 0.035 | * |
| total expression | 16.745 | 0.015 | * |

Note: *P*-values were obtained from 1,000 permutations. Significance codes: * *P* < 0.05; ns = not significant.


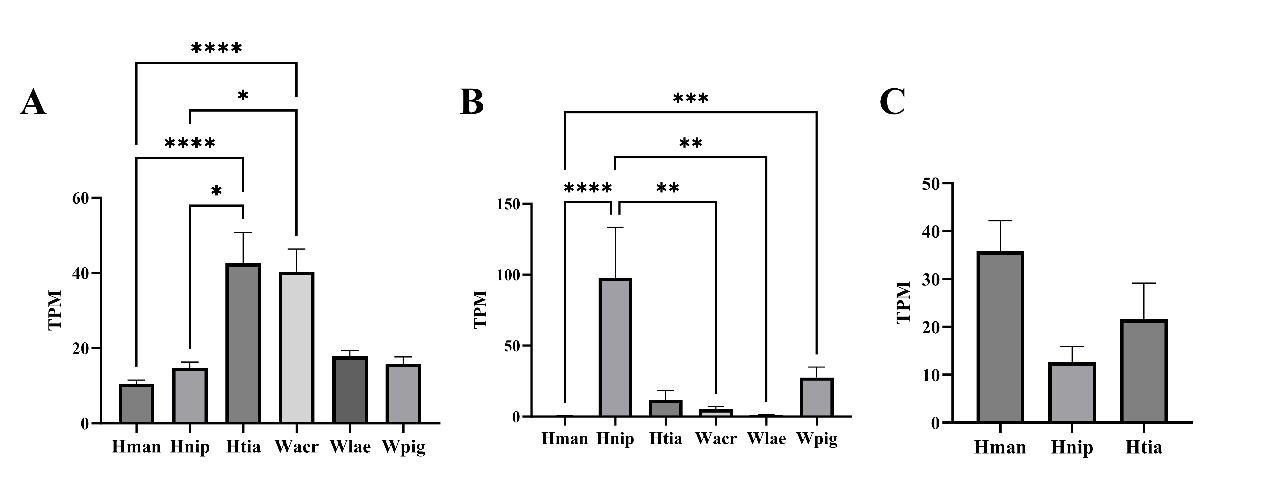
**Supplementary Figure S1.** Comparative expression of hyaluronidase gene family members *hya3*–*hya5* across leech species. (A) Comparison of *hya3* expression; (B) Comparison of *hya4* expression; (C) Comparison of *hya5* expression. Error bars indicate mean ± SEM. **P* < 0.05, ***P* < 0.01, ****P* < 0.001, **** *P* < 0.0001; comparisons without asterisks are not significant.
